# Leveraging Position Bias to Improve Peer Recommendation

**DOI:** 10.1371/journal.pone.0098914

**Published:** 2014-06-11

**Authors:** Kristina Lerman, Tad Hogg

**Affiliations:** 1 USC Information Sciences Institute, Marina Del Rey, California, United States of America; 2 Institute for Molecular Manufacturing, Palo Alto, California, United States of America; University of Cape Town, South Africa

## Abstract

With the advent of social media and peer production, the amount of new online content has grown dramatically. To identify interesting items in the vast stream of new content, providers must rely on peer recommendation to aggregate opinions of their many users. Due to human cognitive biases, the presentation order strongly affects how people allocate attention to the available content. Moreover, we can manipulate attention through the presentation order of items to change the way peer recommendation works. We experimentally evaluate this effect using Amazon Mechanical Turk. We find that different policies for ordering content can steer user attention so as to improve the outcomes of peer recommendation.

## Introduction

The growing volume of content created in online social media and other peer production systems is making it increasingly difficult to identify interesting items. On YouTube alone, over 100 hours of video are uploaded every minute. Which of the many videos are worth watching? Likewise, which of the thousands of new daily articles and comments on the social news web site Reddit are worth reading?

The challenge facing content providers, such as YouTube and Reddit, is identifying items their user communities will find interesting from among the vast numbers of newly created items. If a better item comes along, content providers need to identify it in a timely manner. Providers have addressed this challenge via peer recommendation. Social news aggregators Digg and Reddit, for example, ask users to recommend interesting news items and prominently feature those with the most recommendations. Flickr and Yelp aggregate their users’ opinions to identify top photos and restaurants respectively. By exposing information about the preferences of others, providers hope to leverage collective intelligence [1] to accelerate the discovery of interesting content. In practice, however, peer recommendation often produces “winner-take-all” and “irrational herding” behaviors in which similar items receive widely different numbers of recommendations [2], [3]. Moreover, collective judgements obtained through peer recommendation are biased [4], [5] and inconsistent, with the same items ending up with very different recommendations under virtually the same conditions [2].

While many strategies for aggregating opinions are possible, not all of them are equally effective in peer recommendation. We investigate some popular strategies and evaluate their ability to identify interesting content. We show that some strategies uncover the underlying population preferences for content more quickly and accurately than others. Our approach exploits *position bias*: people pay more attention to items at the top of a web page or a list of items than those below them [6], [7]. A consequence of this bias is the strong effect of presentation order on choices people make. For instance, presentation order affects which items in a list of search results users click on [8]–[10], and the answer they select when responding to a multiple choice question [6], [11]. Thus, a content provider can change how much attention items receive simply by changing their presentation order.

Studying peer recommendation is difficult due to confounding effects. These include heterogeneity of content quality, its changing relevance (novelty), commonality of user preferences (homophily), and social influence (when showing users a summary of prior users’ behavior). Another important effect is history-dependence, which can be due to having different content available at different times or the web site changing the order of presented items based on prior users’ responses. We disentangle some of these effects through randomized experiments on Amazon Mechanical Turk (Mturk), a marketplace for work [12] which is also an increasingly popular experimental platform for behavioral research [13]–[15]. The experiments allow us to determine how some of the strategies used by content providers for ordering items affect the outcomes of peer recommendation. We experimentally evaluate the effect of position bias, in contrast to previous studies of social influence [2], [3], [5], [16]. By leveraging position bias, we can systematically direct user attention so as to improve peer recommendation. Specifically, we demonstrate that ordering items by recency of recommendation generates better estimates of underlying population preferences than ordering them by their aggregate popularity.

Our experiments showed people a list of science stories and asked them to recommend, or vote for, ones they found interesting. We tested five strategies for ordering content, which we refer to as “visibility policies”. The *random* policy presented the stories in a random order, with a new ordering generated for each participant. The *popularity* policy ordered stories by their popularity, i.e., in decreasing order of the number of recommendations they had already received. The *activity* policy ordered stories in chronological order of the latest recommendation they received, with the most recently recommended story at the top of the list. Finally, the *fixed* policy showed all stories in the same order to every study participant, and the *reverse* policy simply inverted that order. There was no adaptive ordering of content in the last two policies. Each study participant was assigned to one of these policies. We refer to participants who successfully completed the task as “users” in our study.

These orderings are common in social media and peer recommendation applications that exploit collective intelligence. For example, the default presentation of news stories shown in Digg’s front page (circa 2009) was by the time of promotion, which corresponds to a fixed ordering, since every user sees the stories in the same order. Digg users could also sort stories by popularity, i.e., by the number of recommendations they received during the last day or week. A Twitter stream, on the other hand, is ordered by activity, because each new retweet of an item (which we treat as a recommendation) appears at the top of a follower’s stream.

We demonstrate that the choice of ordering policy strongly affects the outcome of peer recommendation. We evaluate these outcomes with respect to the following goals: 1) accurately estimate population preferences for content, 2) rapidly and 3) consistently produce the estimates, and 4) focus user attention on highly interesting content. Specifically, we show that ordering items by activity produces more accurate and less variable estimates than ordering items by popularity, a widely-used policy in peer recommendation for aggregating user opinions. On the other hand, popularity-based ordering more effectively focuses attention on more interesting content.

## Results

This section presents the results of our experiments. The methods section describes the experiment procedures in detail.

### Story Appeal

Item “quality” varies significantly, although it is difficult to define or measure [2]. Instead of “quality” we use story appeal, which we define operationally as the likelihood a user who sees a story votes for (recommends) it. We assume that appeal is stable in time, which generally holds for the science stories in our experiments. While our definition of appeal conflates factors related to a story with preferences and motivations of users, it captures the notion that some content is inherently more appealing or interesting to a community. In general, this conditional probability is difficult to measure because it requires knowing both whether a user saw and voted on a story. While votes are readily recorded, views are not readily available, e.g., requiring eye tracking or, for a less precise measure, whether particular content was delivered to the user’s browser. Nevertheless, controlled experiments can measure the average appeal of a story to a user population [2] by, for example, randomizing over possible confounding effects such as the order of the story. After enough people had seen each story, the number of votes they receive will reflect how interesting or appealing people find them.

**Figure 1:**
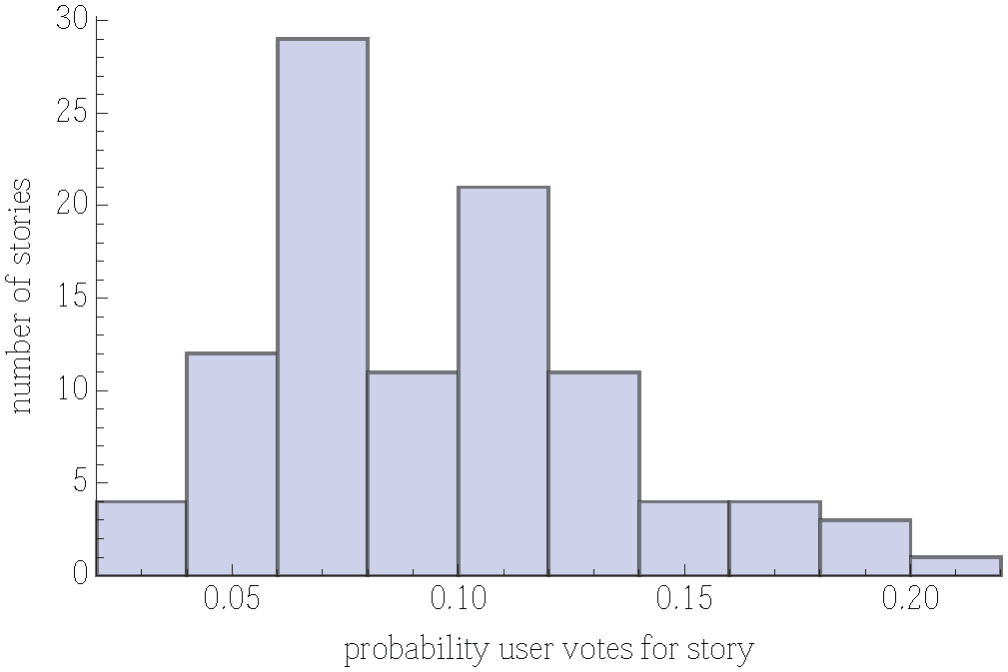
Distribution of story appeal 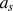, i.e., probabilities users vote on each story under the random order policy.

The random policy in our experiments provides the control for estimating appeal. Specifically, we define the appeal 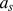 of a story 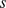 to a population of users as the fraction of users in a sufficiently large sample from that population who vote for 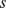. The random policy averages over positions, so 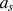 captures the underlying population preferences for stories. Fig. 1 shows that appeal is broadly distributed, varying by about a factor of four among stories.

### Position Bias

**Figure 2:**
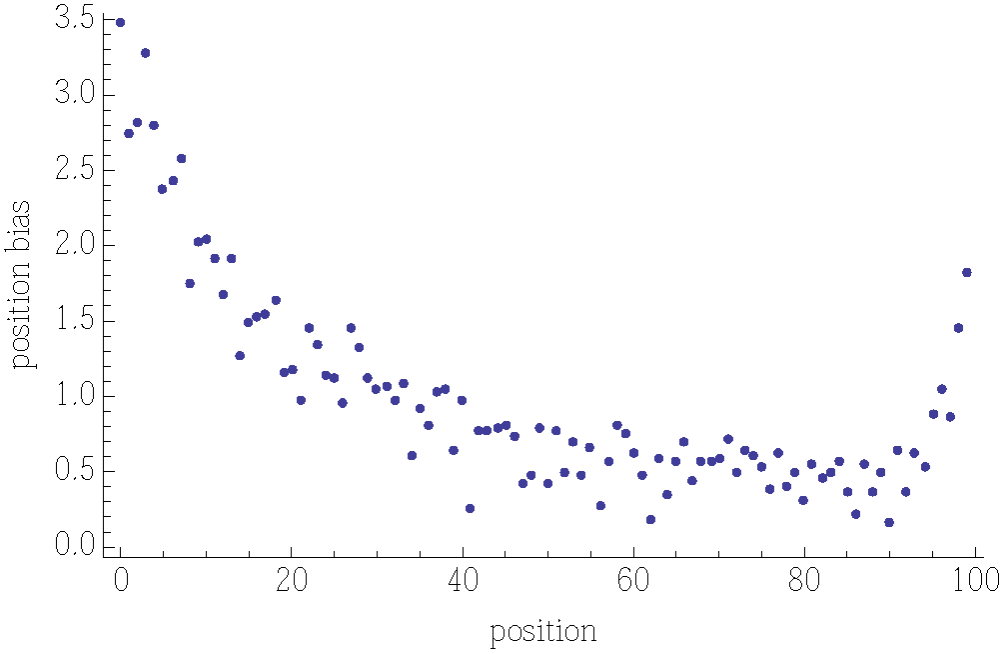
Position bias: variation in votes based on position.

The probabilities for votes on each story (i.e., its appeal) allow estimating the number of votes we would expect at each position in the random policy. Specifically, suppose stories 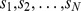 are shown to successive users at position 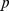. The expected number of votes for these stories is 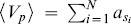. With 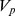 the actual number of votes for these stories, the ratio 
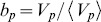
 is the relative increase or decrease in votes for that position compared to average, i.e., position bias. Fig. 2 shows these ratios.

Position bias is quite pronounced: a story at the top of a list gets about five times as much attention as a story lower in the list. This behavior is similar to how users respond to web search results [8]–[10], content in social media (e.g., Digg [17]), and online cultural marketplaces [16], [18]. The moderate increase in votes at the end of the list was observed by Salganik et al. [16], who attributed it to ‘contrarians’, who navigate the list starting from the end. Another possibility is this behavior results from strategic decisions made by participants to give an impression that they had inspected all stories.

### Votes and Appeal

**Figure 3:**
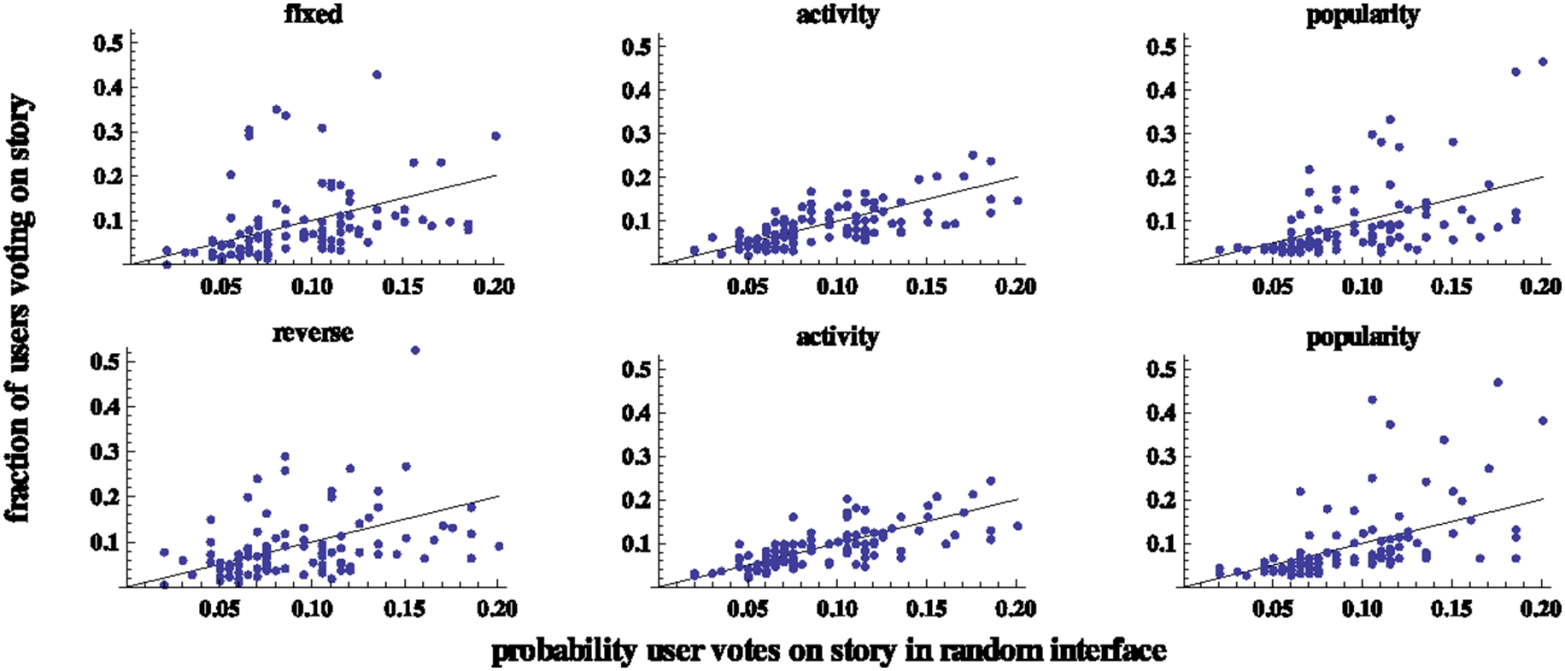
Fraction of users voting for a story vs its appeal 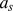 under different policies for ordering stories. The lines are the expected number of votes per user based on the random policy.

Fig. 3 shows the variation in votes on stories, compared to the random policy. The activity policy, by continually moving recommended stories to the top of the list, divides user votes roughly in proportion to their appeal. The popularity policy is much more variable, both among stories with similar appeal and between repeated experiments. The fixed policy focuses user attention on the same stories, leading to a large deviation from their appeal. Similarly, all users in the reverse policy see the stories in the same order, which also leads to a large deviation. Specifically, the fixed and reverse policies have correlations between votes and appeal of 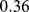 and 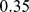, respectively. Both parallel worlds for the activity policy have larger correlations, 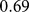 and 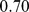, while the popularity policy is intermediate between activity and fixed, with correlations 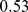 and 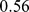 in the parallel worlds experiments. These correlations are statistically significant, with 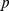-values less than 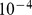 in all cases according to the Spearman rank test for zero correlation. The activity policy leads to, on average, higher correlation between votes and appeal than the other policies. Since an item’s popularity is often used as a proxy for how appealing it is to a user population, the activity policy is better for evaluating items.

**Figure 4:**
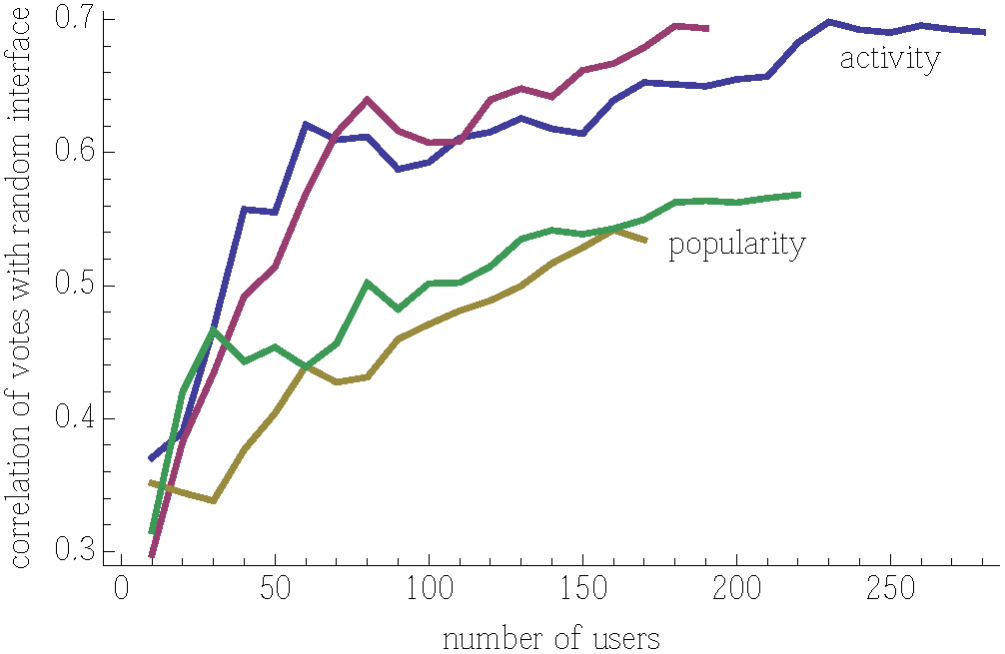
Correlation between number of votes each story receives and its appeal as a function of number of users voting.

Next we examine how quickly the policies estimate appeal. While both popularity and activity policies quickly converge to their estimates, the popularity policy may be slow to respond to changing user interests. This is because after the first 50 or so users, the popularity policy becomes a (nearly) fixed ordering, with stories near the top of the list accumulating votes more rapidly than other stories, making it difficult for a new, more appealing story to reach the top position. One measure of the responsiveness of a policy is how rapidly the number of votes approaches that expected from the stories’ appeal. Fig. 4 shows this behavior. Repeated experiments with each policy give consistent behavior. Activity converges more rapidly, and to a higher correlation with appeal, than popularity. The final values of the correlations correspond to those for all votes, discussed with Fig. 3.

### Inequality of Outcomes

Variations in the distribution of attention produced by different orderings lead to large differences in the number of votes stories receive, i.e., their popularity. Since stories differ in appeal, when attention is distributed uniformly (as in the random policy) we expect votes to vary in proportion to their appeal. Orderings that direct user attention toward the same stories will result in greater inequality of popularity.

We quantify the variation in popularity of stories by the Gini coefficient, a measure of statistical dispersion:
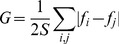
(1)where 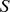 is the number of stories and 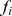 is the fraction of all votes that story 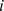 receives, so 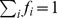. In our experiments, 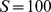.

**Figure 5:**
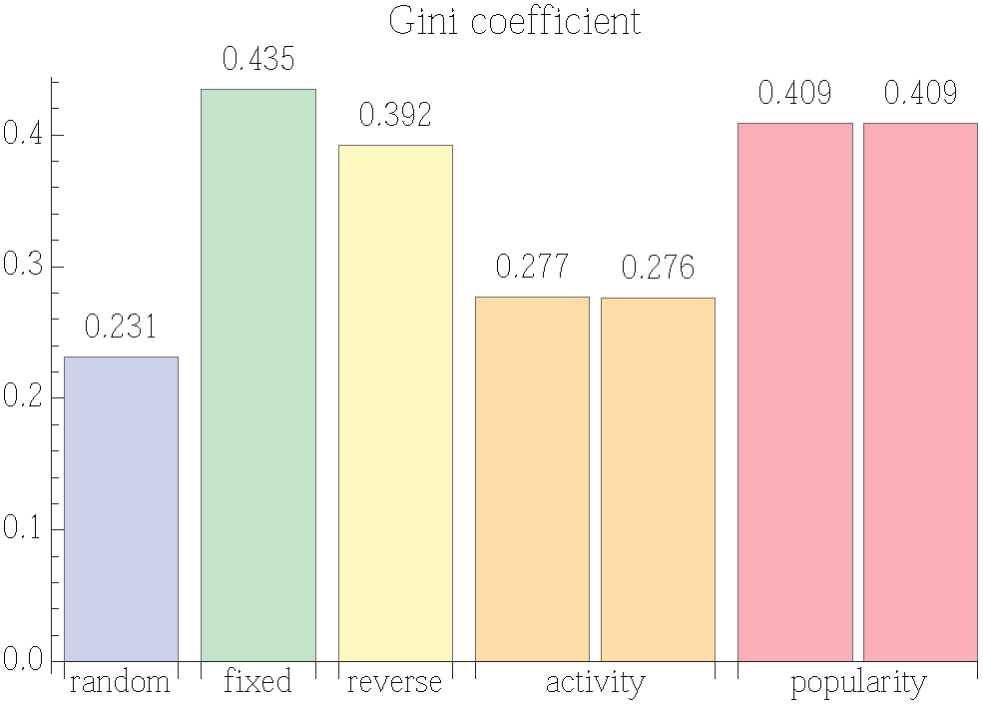
Gini coefficient showing inequality of the total votes received by items in different policies.

Fig. 5 shows the values of the Gini coefficient in our experiments. In the random policy, the fraction 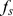 is, by definition, the appeal 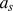 for that story. Thus the value for the random policy indicates the inequality expected solely from the variation in story appeal. The activity policy results in slightly more inequality than would be expected from the inherent differences in story appeal. On the other hand, a policy that shows stories in a fixed order focuses attention on the same most visible stories, leading to a large inequality in the distribution of votes. This is the case for the fixed and reverse policies. This observation also explains the large inequality in the popularity policy because its story order essentially stops changing after 50 users make recommendations. Thus, for subsequent users its position bias is similar to that of a fixed policy. As a consistency check, the two parallel worlds for each of the activity and popularity policies give the same Gini coefficients. Nevertheless, the particular stories receiving the most votes differ between the two worlds, especially for the popularity policy.

For the policies without history dependence (i.e., random, fixed and reverse), we can assess the significance of the different Gini coefficients with a permutation test. Specifically, to compare two policies under the null hypothesis that they do not differ in how user choices contribute to inequality, we randomly permute the users in those experiments between the policies, while keeping the same *number* of users assigned to each policy. From this permutation, we compute the resulting difference in Gini coefficients. Repeating this evaluation many times gives an estimate of how the difference would vary if user behavior was the same in the two policies. Comparing this variation with the actual difference in Gini coefficient between those two policies indicates how likely that observed difference could arise under the null hypothesis. We use this method to compare each pair of the three policies (random, fixed and reverse), using 100 permutations for each pair. In all cases, the observed difference in Gini coefficient is larger than the differences from all these permutations, indicating the differences are significant with 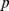-value below 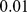.

This permutation test does not apply to the policies with history dependence (i.e., activity and popularity), since the presentation of stories depends on the actions of previous users. Instead, repeating the experiments (i.e., parallel worlds) gives independent estimates of the Gini coefficient for these policies. The small differences in Gini coefficients between the parallel worlds for each policy suggests the popularity policy leads to greater inequality than the activity policy.

### Predictability of Outcomes

Fig. 3 shows votes under the popularity policy have larger variation than those under the activity policy, particularly for high-appeal stories. Moreover, comparing the outcomes of parallel worlds experiments shows much larger consistency between worlds for the activity policy. For instance, the top quartile of stories (those with 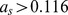) have correlations 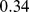 and 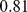 between votes in the parallel worlds for popularity and activity policies, respectively. This large a difference in correlation is unlikely to arise if in fact these two policies had the same correlations between parallel worlds (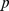-value 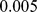 with the Spearman rank test). Moreover, the pattern of votes in the two parallel worlds for popularity is consistent with no correlation between the worlds (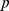-value 0.2 with Spearman rank test). On the other hand, zero correlation is unlikely for the activity policy (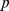-value 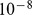). Thus outcomes are more predictable for the activity policy: a given high-appeal story is more likely to get a similar number of votes if repeated with a new group of users. Popularity, on the other hand, is less consistent due to the amplification of the effects of early votes through its “rich get richer” behavior.

In contrast with stories in the top quartile, these two policies have no significant difference in correlation for the less appealing stories: those stories receive similar, low numbers of votes in both parallel worlds for each policy.

### Focusing Attention on Appealing Items

How well do the visibility policies focus user attention on appealing stories? This is an important measure of user experience in peer recommendation systems: showing users appealing stories indicates to those users the site has interesting content, making it more likely the users will return to the site [19].

Web users typically view only a fraction of the available content, starting from the top of the list of items. Thus one measure of user experience is the appeal of the stories they are most likely to view, i.e., those near the top of the list. We quantify this aspect of user experience by how well the policy delivers high-appeal stories to early positions in the list of stories shown to a user. As a specific example, we examine the first 20 positions and measure the fraction of those positions containing stories whose appeal is among the top 20% of stories (as measured in the random policy).

**Figure 6:**
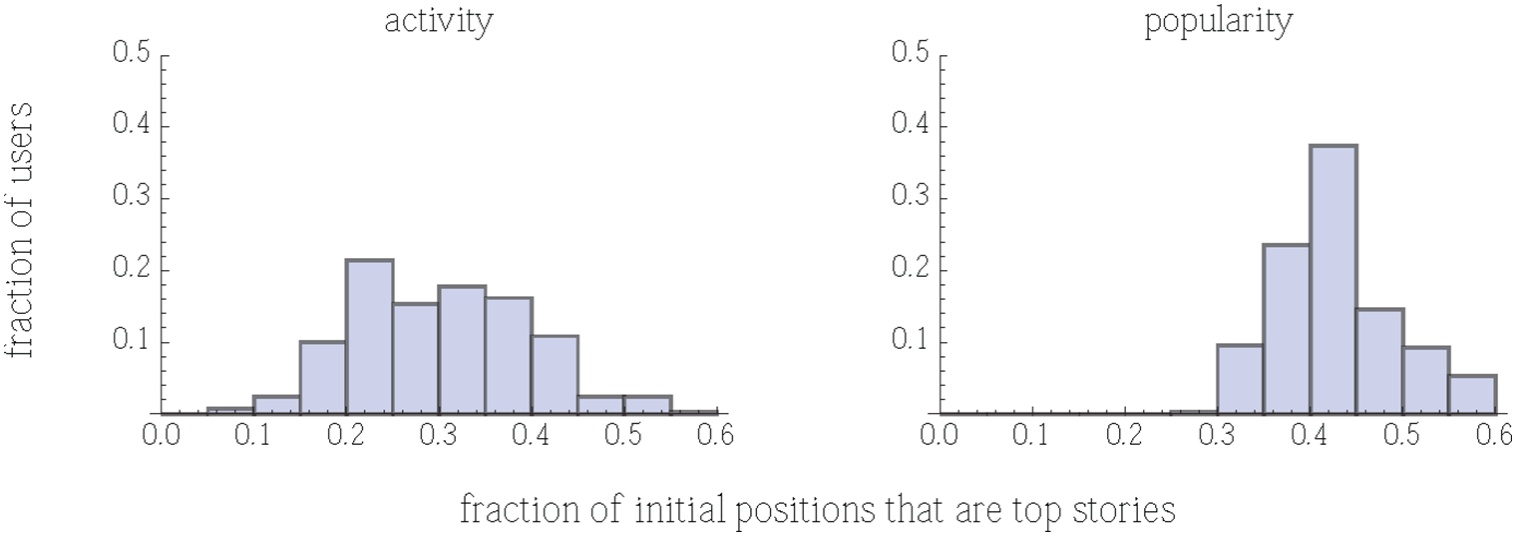
Distribution of fraction of the first 20 stories shown to a user that are among the most-appealing 20% of stories. Under the popularity policy, for most users at least 40% of the initial stories are among the most-appealing stories, whereas under the activity policy, most users see fewer than 40%. These histograms do not include the first 50 users in each experiment, to avoid the initialization phase of the policies.

Fig. 6 shows the resulting distributions for users assigned to the activity and popularity policies. By this measure of user experience, the activity policy has lower average fraction and is more variable among users than the popularity policy. In other words, users assigned to the activity policy tend to see fewer top stories than users assigned to the popularity policy. Moreover, the high variability under the activity policy means a significant fraction of users are likely to see very few top stories. For comparison, users assigned to the random policy will likely see about 20% of the top stories, which is even less than the activity policy.

## Discussion

Our findings demonstrate that the ordering of items significantly affects the outcome of peer recommendation. The differences in outcomes stem from human cognitive biases, specifically the position bias that results in people paying more attention to items appearing near the top of the list. These items have high visibility, since it takes little effort to discover them. The more effort required to find an item, the less attention it will receive. While this bias cannot be altered, we can control which items people pay attention to simply by changing their position in the list of items.

Visibility policies differ in how well they fulfill the goals of peer recommendation described in the introduction. Clearly, random policy is best for unbiased estimates of preferences. However, since a small fraction of user-generated content is interesting, users will mainly see uninteresting content under the random policy. As a consequence, they may then form an impression that the site does not provide anything of interest and fail to return. Unlike the random policy, the popularity policy does not accurately estimate preferences, since small early differences in popularity may be amplified via a “rich get richer” effect. As a result, item ordering quickly becomes fixed, which leads to greater inequality and less consistency. On the other hand, the popularity policy emphasizes highly appealing content for users better than the random policy does. In contrast, the recency condition of the activity policy leads to more robust estimates of underlying population preferences than ordering by popularity. It was second only to the random policy in how well the observed popularity correlated with user interests in items, and also produced less variable, more predictable outcomes. While the activity policy was not as effective as the popularity policy at focusing user attention on appealing content, it was better than the random policy. The activity policy is also a good choice for time critical domains, where novelty is a factor, since continuously moving items to the top of the list can rapidly bring newer items to users’ attention. In summary, the choice of ordering allows steering peer recommendation toward a desired goal, such as accurately estimating appeal or highlighting interesting content for users visiting the web site.

Beyond peer recommendation, position bias also affects the performance of social media, discussion forums, online markets, and crowdfunding sites. Specifically, the amount of attention a message receives in social media is largely determined by its position in the user’s stream, and this affects the ease with which the message spreads [17], [20]. By directing user attention to certain messages, a social media site can selectively enhance their spread. Crowdsourcing applications which require users to select tasks or items from a list can similarly manipulate individuals’ attention to drive human computation in a particular direction. In online discussion forums, user attention can be directed so as to improve the performance of distributed moderation. Current moderation schemes can give messages unfairly low scores, because early negative scores reduce their visibility and prevent them from receiving the attention needed for a fair evaluation [4].

Quantitative understanding of position bias is important from the design perspective, as it allows for more accurate and robust estimation of how interesting some content is to a user population. For instance, a web site could estimate content appeal from the responses of an initial cohort of users, and then place content with the highest estimated appeal in the most visible positions to improve user experience. The web site could also adjust its presentation method dynamically to adapt to changing user preferences and content novelty.

Our study did not directly examine social influence, since users were not shown the number of votes stories received. For influence to occur, a social signal has to be present, but even then, the individual first has to discover the item before he or she can be affected by this signal. Hence, an item’s visibility, which affects how easily it can be discovered, plays a big role in how popular it will become.

Our experiments are similar in design to those of Salganik et al. [2], [21], which examined why some cultural artifacts become vastly more popular than others, and why their popularity is largely unpredictable. The studies asked participants to rate songs by unknown bands. Songs were presented either in random order (*cf* random policy) or sorted by popularity (*cf* popularity policy). Salganik et al. found that sorting by popularity resulted in more unpredictability and greater inequality of popularity. Moreover, providing a signal of popularity, by showing participants how popular songs are, further increased inequality and unpredictability. They attributed both effects to social influence. In contrast, our study suggests that inequality and unpredictability of popularity could arise even in the absence of social influence, since biases in perception lead users to pay more attention to items near the top of the list. If those items are already the most popular ones, this creates a “rich get richer” effect that amplifies their popularity. A re-examination of Salganik et al.’s experimental data [18] showed that a song’s position in the list can explain much of its near-term popularity. This is encouraging, as it suggests that knowing an item’s visibility can help predict its future success.

## Methods

University of Southern California’s Institutional Review Board (IRB) reviewed the experiment design and classified it as “non-human subjects research.” Our experiments were published as tasks (HITs) on Amazon Mechanical Turk, which allowed us to recruit study participants from a large pool of workers. Workers who accepted the task were shown the following instructions: “We are conducting a study of the role of social media in promoting science. Please click ‘Start’ button and recommend articles from the list below that you think report important scientific topics. When you finish, you will be asked a few questions about the articles you recommended. (Please remember, once you finish the job, system won’t allow you to do it again).” They were paid $0.12 for completing the experiment and each person was allowed to do the experiment only once. The pay rate was set low to make the task less attractive to workers attempting to game Mturk and is comparable to similar tasks in other research studies [12], [14]. Although we paid people to vote, we assume their behavior is similar to that in recommendation systems. This assumption is validated by the growing body of work using Mturk for behavioral research [13]–[15].

**Figure 7:**
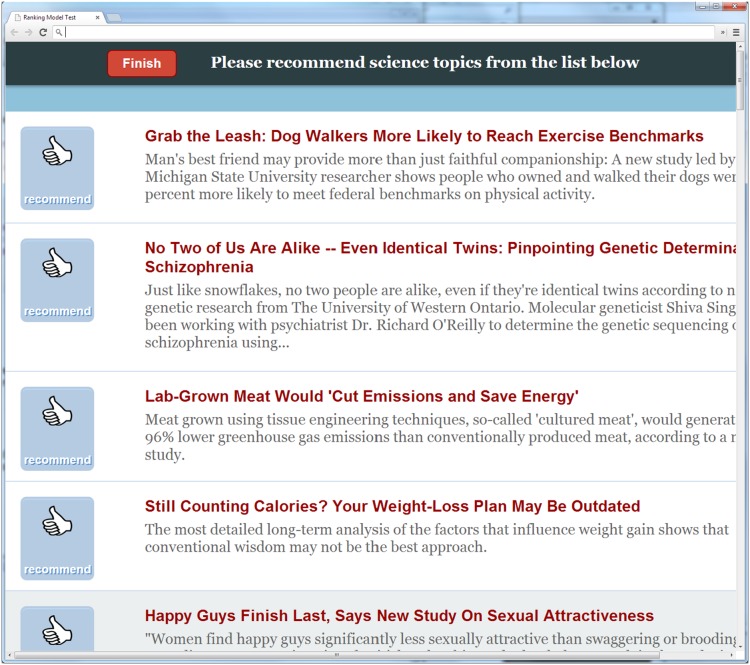
Screenshot of a web page shown to participants.

We showed the participants a list of one hundred science stories, drawn from the Science section of the New York Times and science-related press releases from major universities (sciencenewsdaily.com). Stories were delivered to the browser in a single page, as illustrated in Fig. 7. The list was sufficiently long to require them to scroll to see all stories. Each story contained a title, a short description, and a link to a page where the person could read the full story. Participants could choose to recommend a story based on the short description or click on the link to view the full story. We recorded all actions, including recommendations and URL clicks, and the position of all stories shown to each participant. When a person recommended a story, the recommend button changed color to indicate that story was recommended. The experiment did not allow participants to undo their recommendations: subsequent clicks of the recommend button brought up a message box reminding participants to recommend a story only once. Although participants were not told ahead of time how many stories to recommend, if they tried to finish the task before making five actions (either recommendations or URL clicks), a message box prompted them to make five recommendations.

Upon finishing the task, participants were asked to name two important themes in the stories they recommended and solve a simple arithmetic question. Only those who correctly answered the arithmetic question were considered to have completed the task and paid. There were nine participants with corrupted session data, which were not included in the analysis. Of the 4,007 workers who accepted the task, only 2,643 completed it. Further, to ensure data quality (see below), we ignored recommendations made by participants who recommended more than 20 stories. The recommendations made by the remaining 1518 people (i.e., users) were saved in a database and are summarized in Table 1. Only these recommendations were used in analysis. Recommendations data are available from the authors upon request.

**Table 1:** Summary of experiments.

policy	users	votes	avg.	std. dev.
random	199	1873	9.4	4.7
fixed	217	1978	9.1	4.8
reverse	221	1999	9.0	4.7
activity	286 & 193	2586 & 1764	9.0 & 9.1	4.5 & 4.6
popularity	174 & 228	1570 & 2162	9.0 & 9.5	4.7 & 4.5
total	1518	13932		

Number of participants and votes made under different visibility policies. The history-dependent orderings (activity and popularity) each have two independent experiments. The last two columns give the average and standard deviation of number of votes per user.

### Visibility Policy

Our experiments allow controlling the presentation of stories and monitoring URL clicks and recommendations, but not tracking which stories are viewed. We studied the visibility policies described above. In each experiment, stories initially had no recommendations, and the popularity and activity policies used the same story order as the fixed policy. The fixed order was also used to break ties in the popularity and activity policies. The random policy was our control condition.

As our focus is on the effect of visibility, we eliminate any confounding effect of social influence [2] by not showing the number of recommendations the stories received or disclosing the method by which we ordered stories. We tested the reproducibility of results for the history-dependent activity and popularity policies by creating “parallel worlds” experiments [2], in which we ran two instances of each policy starting from the same initial conditions.

### Data Quality Control

Amazon Mechanical Turk is an appealing platform for studies of human behavior. However, a major challenge for using Mturk is ensuring data quality [14], because some workers, i.e., spammers, fail to exert the effort necessary to evaluate stories. Instead they do the least work to get paid, e.g., click on the first story or on every story.

We used a multi-step strategy to reduce spam. First, we selected workers using qualifications provided by Mturk: they lived in the US, had completed at least 500 tasks on Mturk, and had a 90% or above approval rate. In addition, after workers finished recommending stories, we asked them to solve a simple arithmetic problem. A new problem was generated after an incorrect answer, preventing them from finding the solution by exhaustive search.

**Figure 8:**
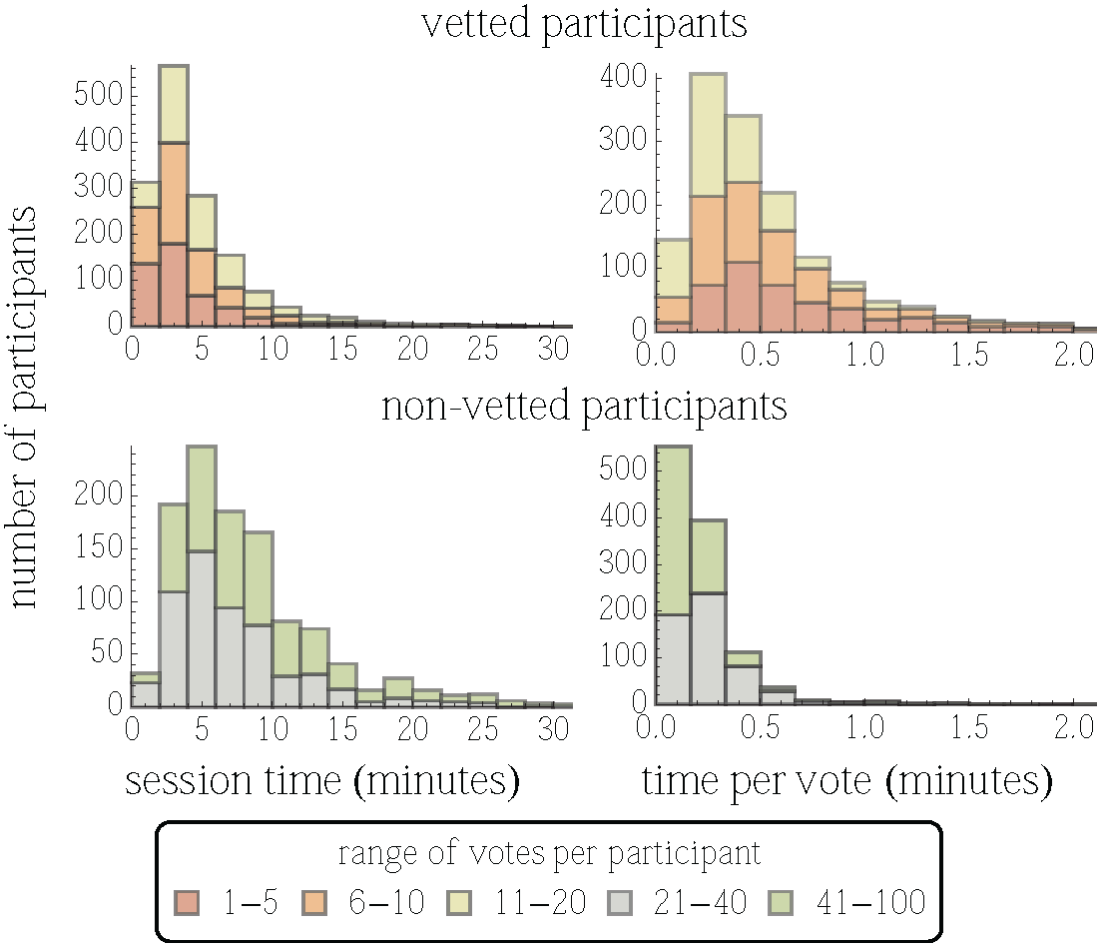
Distribution of session time (left column) and average time per vote (right column) for vetted and non-vetted participants. Participants are grouped according to their activity, i.e., number of votes, with each group (indicated by a colored bar) containing about 500 people. A few people with longer session times and times per vote are not included in the plots.

In spite of our selection process, we found large apparent variation in motivation. Some participants appeared not to make a serious effort in evaluating stories and simply recommended most or all of the stories. To exclude such people, our vetting procedure accepted only the recommendations from participants who recommended at most 20 stories. Such vetted participants were the users in our study. They generally spent more time evaluating each story. Fig. 8 shows the distribution of session times (excluding the time required to read instructions and do the post-survey) and the average time taken by participants to recommend a story. While non-vetted participants spent a little more time on the task, it took a typical vetted participant (voting on at most 20 stories) 25 seconds to recommend a story, while a non-vetted participant required fewer than 10 seconds. These differences are statistically significant (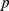-values less than 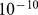 with Mann-Whitney tests). In addition, the rate at which participants clicked URLs, an action not required by the task but which suggested motivation, was higher for vetted (27%) than non-vetted participants (22%), with 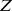-test indicating these proportions are different (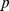-value 0.01). Although the choice of the 20-recommendation threshold is somewhat arbitrary, timing results and URL clicks indicate that it appropriately weeded out unmotivated participants.
